# MicroRNA regulatory pathway analysis identifies miR-142-5p as a negative regulator of TGF-β pathway via targeting SMAD3

**DOI:** 10.18632/oncotarget.12229

**Published:** 2016-09-24

**Authors:** Zhaowu Ma, Teng Liu, Wei Huang, Hui Liu, Hong-Mei Zhang, Qiubai Li, Zhichao Chen, An-Yuan Guo

**Affiliations:** ^1^ Hubei Bioinformatics and Molecular Imaging Key Laboratory, Department of Bioinformatics and Systems Biology, Key Laboratory of Molecular Biophysics of the Ministry of Education, College of Life Science and Technology, Huazhong University of Science and Technology, Wuhan, 430074, China; ^2^ Laboratory of Neuronal Network and Brain Diseases Modulation, School of Medicine, Yangtze University, Jingzhou, Hubei, 434023, China; ^3^ Institute of Hematology, Union Hospital, Tongji Medical College, Huazhong University of Science and Technology, Wuhan 430022, China

**Keywords:** microRNA, regulatory network, cancer, miR-142-5p, TGF-β pathway

## Abstract

MicroRNAs (miRNAs) are non-coding RNAs with functions of posttranscriptional regulation. The abnormally expressed miRNAs have been shown to be crucial contributors and may serve as biomarkers in many diseases. However, determining the biological function of miRNAs is an ongoing challenge. By combining miRNA targets prediction, miRNA and mRNA expression profiles in TCGA cancers, and pathway data, we performed a miRNA-pathway regulation inference by Fisher's exact test for enrichment analysis. Then we constructed a database to show the cancer related miRNA-pathway regulatory network (http://bioinfo.life.hust.edu.cn/miR_path). As one of the miRNAs targeting many cancer related pathways, miR-142-5p potentially regulates the maximum number of genes in TGF-β signaling pathway. We experimentally confirmed that miR-142-5p directly targeted and suppressed SMAD3, a key component in TGF-β signaling. Ectopic overexpression of miR-142-5p significantly promoted tumor cell proliferation and inhibited apoptosis, while silencing of miR-142-5p inhibited the tumor cell proliferation and promoted apoptosis *in vitro*. These findings indicate that miR-142-5p plays as a negative regulator in TGF-β pathway by targeting *SMAD3* and suppresses TGF-β-induced growth inhibition in cancer cells. Our study proved the feasibility of miRNA regulatory pathway analysis and shed light on combining bioinformatics with experiments in the research of complex diseases.

## INTRODUCTION

MicroRNAs (miRNAs), an abundant class of small non-coding regulatory RNAs, mediated translation inhibition or mRNA degradation by binding to the 3′ untranslational region (3′UTR) of target mRNAs [1]. Mounting evidence has shown correlations between various human cancers and miRNAs because of their mutations or the aberrant expressions [2, 3]. MiRNAs may function as tumor suppressors or oncogenes, depending on the cellular function of their targets [4]. Thus, aberrant miRNA expression can be regarded as a common feature of cancers; identification of these dysregulated miRNAs and their respective targets may provide potential biomarkers for cancer diagnostics and new therapeutic strategies against cancers [5, 6].

A growing number of studies have demonstrated that one miRNA may regulate a few hundreds of genes on average [7]. Currently, several studies predicted the functions of miRNAs based on the functional enrichment analysis of their target genes. miRGator and DIANA-mirPath provided the statistically enriched Gene Ontology functions and KEGG/GenMAPP/BioCarta pathways [8, 9]. Another comprehensive analysis established a dictionary on miRNAs and their putative target pathways and uncovered that differentially expressed genes in cancers were enriched with targets of certain miRNAs [10]. Qiu et al. developed a miR2Gene tool to discover the pattern of gene and pathways by enrichment analysis of their miRNA regulators [11]. Recent studies performed an integrated analysis of miRNA and gene expression profiles to miRNA-gene regulatory network in complex diseases [12, 13]. However, the combination of comprehensive prediction and experimental validation was seldom in miRNA regulatory pathway.

The transforming growth factor-β (TGF-β) signaling pathway plays pivotal roles in multiple physiological and disease processes [14, 15]. TGF-β is a pleiotropic cytokine, exerts its effect on gene expression through the transcription factors known as SMAD proteins. After binding of TGF-β by its heterodimeric receptors TGF-β receptor I and II, the receptors phosphorylate SMAD2 and SMAD3, which then associate with SMAD4 and translocate to the nucleus [16]. Over-activity of TGF-β signaling has been linked to various diseases and exerts a complicated role. Initially, it is a tumor suppressor that inhibits the growth of cells and induces apoptosis. However, at later stages of tumor progression, TGF-β acts as a tumor promoter which correlates to increased invasiveness and metastasis [17]. Many miRNAs were examined to target the TGF-β pathway in different cancers, such as miR-34a in glioblastoma, miR-199a in gastric cancer, miR-142-3p in lung cancer, and miR-146a in acute promyelocytic leukemia [18–22]. However, to date, the function of miR-142-5p in TGF-β pathway remains elusive.

In this study, we performed a miRNA regulatory pathway inference and identified several cancer-related networks between miRNAs and pathways. To verify the reliability of regulatory network inference, we experimentally confirmed that miR-142-5p indeed regulated the canonical TGF-β signaling pathway via targeting SMAD3. Furthermore, experimental validation indicated that miR-142-5p promotes cell proliferation by attenuating TGF-β mediated inhibitory effects in tumor cells. Consequently, this study enhanced the understanding of miRNA regulatory role in tumors and also provided a feasible approach with bioinformatics guidance in complex diseases.

## RESULTS

### MiRNA pathway enrichment analysis and miR-pathway database construction

To infer the regulation between miRNAs and pathways in different tumor types, firstly we collected the miRNA targets from multiple databases by both prediction and experimental validation (see Methods and Supplementary Figure S1). We used the data of 20 tumor types from TCGA (http://firebrowse.org/), which had miRNA and mRNA expression data in both case (tumor) and normal (tumor adjacent) samples. The differentially expressed miRNAs and mRNAs were filtered for further target prediction by cutoffs in methods. Then, the collected miRNA target pairs with a negative correlation between miRNA and mRNA were considered as true miRNA-target pairs. We applied one-tail Fisher's exact test to explore the enrichment analysis of miRNA target genes in each BioCarta pathway and tumor type (Supplementary Table S1). The predicted method provides an accurate *P*-value for the enrichment significance and has been widely used [9, 23]. Thus, we obtained the regulation of miRNA-pathway across 20 tumor types (*P* < 0.05). A total of 19588 miRNA regulatory pathway pairs showed negative correlations in 20 cancers (Supplementary Table S2).

**Figure 1:**
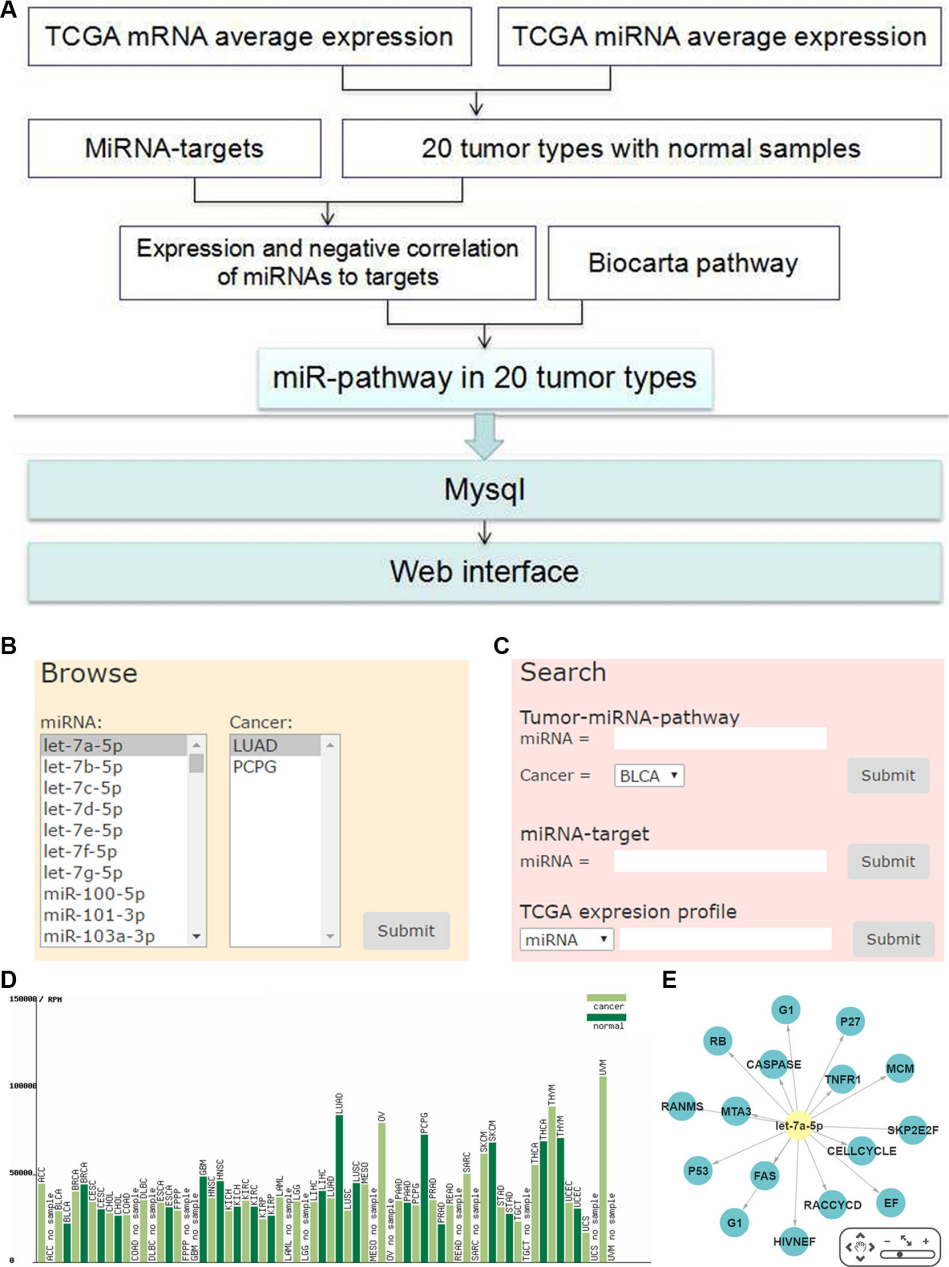
Work flow and web interface of miRNA-pathway regulation (**A**) The overview schema of work flow. (**B**) Browse of miRNA-pathway of tumor types. (**C**) Search for miRNA-pathway of tumor types. (**D**) TCGA expression of miRNA or mRNA in each tumor type. (**E**) Network of miRNA pathway.

We constructed a database with user-friendly web interface to display the miRNA pathway regulation and their expressions (http://bioinfo.life.hust.edu.cn/miR_path) in the 20 tumor types. The database provides search, browse and download function for the miRNA-pathway data. Users can search miRNA regulating pathways, miRNA target genes and the expression profiles of miRNAs and genes in TCGA (Figure 1B–1E). Taken together, we provided a preeminent cancer research resource by combining the differentially expressed miRNAs/genes with miRNA regulatory pathway analysis.

### Tumor differentially expressed miRNAs and their regulating pathways

**Figure 2:**
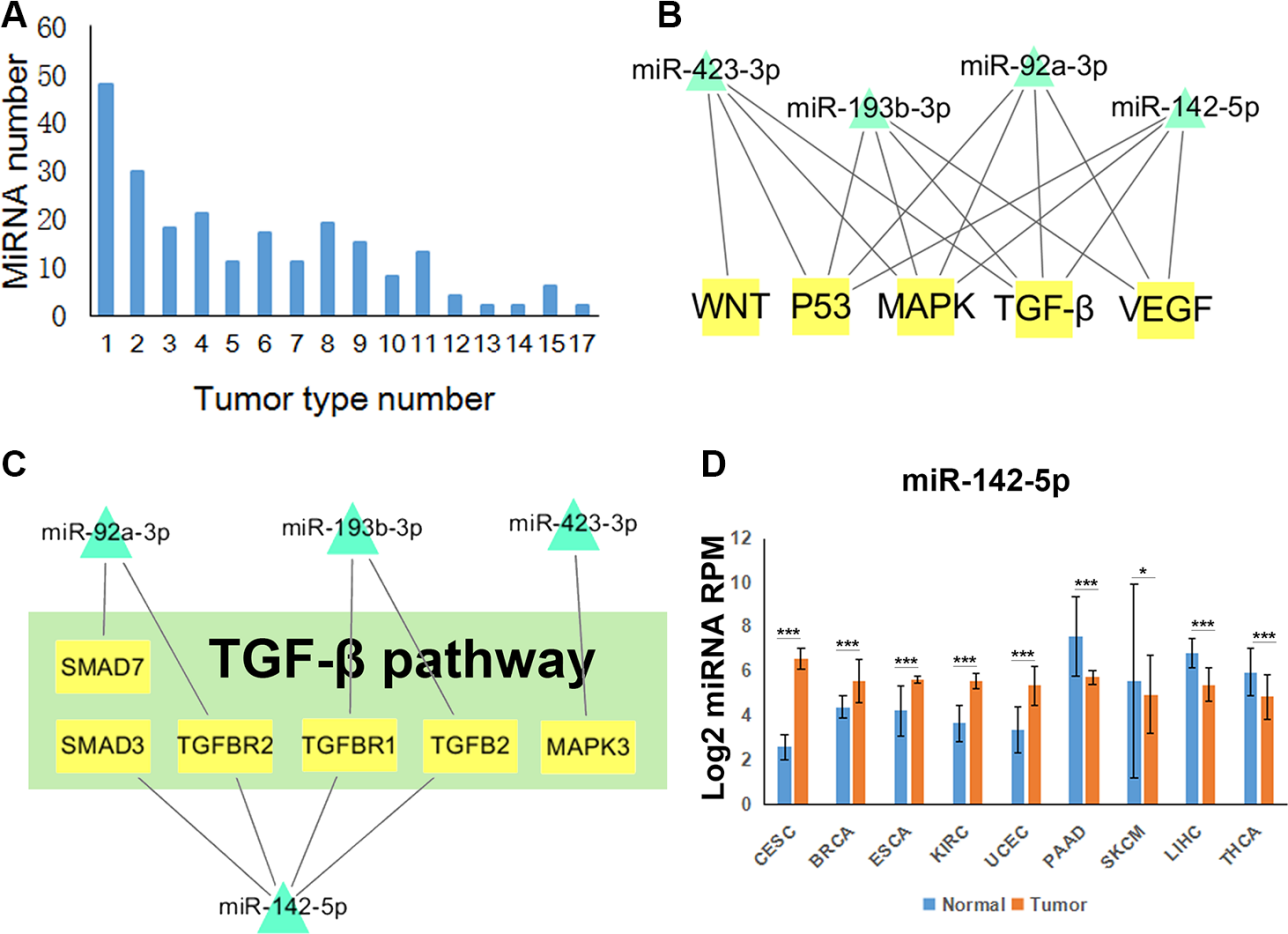
Tumor-related miRNA regulatory pathways (**A**) The distribution of differentially expressed miRNAs in different number of tumor types. (**B**) Network of miR-92a-3p, miR-142-5p, miR-193b-3p and miR-423-3p regulating five of the six pathways. (**C**) miRNA target network in TGF-β signaling pathway regulated by those four miRNAs. (**D**) The expression profiles of miR-142-5p normalized by log2 in nine tumor types. *T* test, *P < 0.05, **P < 0.01, ***P < 0.001. (CESC: Cervical squamous cell carcinoma and endocervical adenocarcinoma, BRCA: Breast invasive carcinoma, ESCA: Esophageal carcinoma, KIRC: Kidney renal clear cell carcinoma, UCEC: Uterine Corpus Endometrial Carcinoma, PAAD: Pancreatic adenocarcinoma, SKCM: Skin Cutaneous Melanoma, LIHC: Liver hepatocellular carcinoma, THCA: Thyroid carcinoma).

By miRNA-pathway enrichment analysis of 20 tumor types, we obtained 227 miRNAs that were differentially expressed in at least one cancer with regulated pathways. There are 48 miRNAs differentially expressed in only one tumor type and about half miRNAs were differentially expressed in less than 5 tumor types (Figure 2A). Significantly, 37 miRNAs are differentially expressed in more than 10 tumor types (Supplementary Figure S2). Some of these miRNAs have been supported by many previous studies. For example, miR-17-5p, miR-21-5p and miR-146b-5p are differentially expressed in a large number of cancer types, and they were reported to target TGF-β signaling pathway in blood and solid tumors [24–27]. MiR-183-5p was found expressed differently in 17 tumor types, and was reported to inhibit Wnt/β-catenin signaling pathway [28]. Most of these miRNAs had been well studied in many cancer types. Thus, we paid attention to miRNAs that were differentially expressed in less than 10 tumor types.

To display cancer-related miRNA regulatory pathways, we chose six cancer-related pathways, including MAPK, NF-κB, TP53, TGF-β, VEGF and WNT signaling pathways [29], which are both in BioCarta and KEGG. Combined the regulations of miRNAs and the six pathways in 20 tumor types, we obtained a comprehensive network (Supplementary Figure S3). We observed that most of the miRNAs merely regulated one or two pathways. However, miR-92a-3p, miR-142-5p, miR-193b-3p and miR-423-3p regulated five of the six pathways (Figure 2B). TGF-β pathway is an important pathway in cancer and we found that miR-142-5p regulated maximum target genes in TGF-β pathway (Figure 2C). Furthermore, the expression profile showed that miR-142-5p is up-regulated or down regulated in nine tumor types (Figure 2D). Collectively, these results indicated that miR-142-5p could influence cancer by targeting TGF-β pathway and could have different functions in different tumor types.

### miR-142-5p regulates the TGF-β signaling pathway by targeting SMAD3

The cancer-related miRNA regulatory pathway analysis suggested that miR-142-5p regulates the TGF-β pathway in several cancers. Our miRNA-pathway analysis identified that miR-142-5p potentially regulated four genes in the TGF-β pathway, which is involved in homeostasis and tumor progression. Thus, we focused our further studies on the function of miR-142-5p in cancer by targeting the TGF-β pathway.

**Figure 3:**
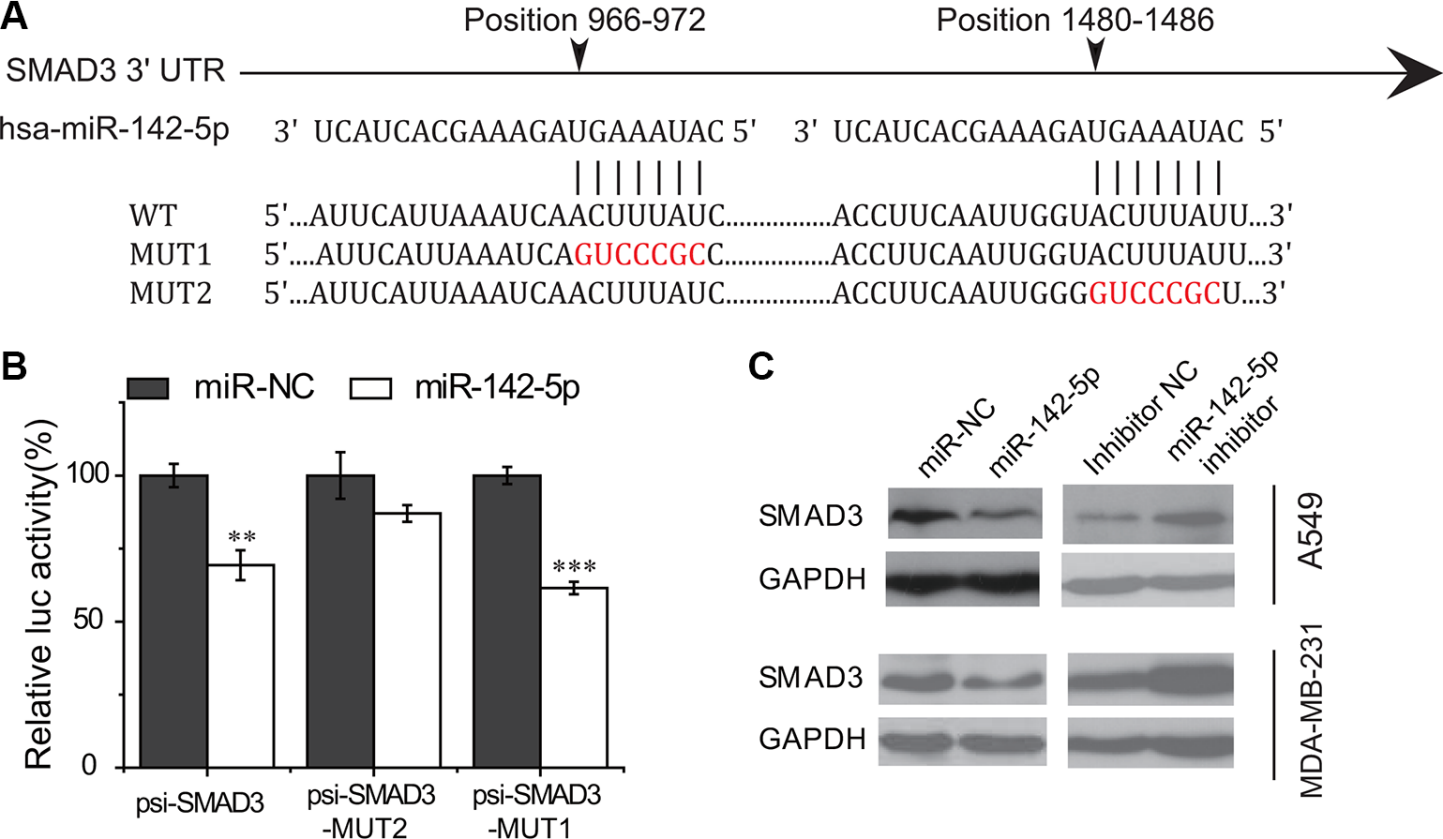
miR-142-5p directly targets SMAD3 (**A**) Sequence alignment of putative miR-142-5p binding sites in the SMAD3 3′ UTRs. WT: wild type; MUT1/2: mutant site 1/2. (**B**) Activity of luciferase gene linked to the 3′ UTR of SMAD3 mRNA. psi-SMAD3-MUT1/2 are the mutated site1/2 of SMAD3. The mutated site 2 of SMAD3 (psi-SMAD3-MUT2) has no effects on miR-142-5p binding. Data represent means and s.e of three experiments performed in triplicate and normalized to control RNA. Data in histograms are represented as mean ± SD. *T* test, **P < 0.01, ***P < 0.001 compared with the control RNA. (**C**) Immunoblotting assays were used to analyze the expression of SMAD3 in A549 and MDA-MB-231 cells transfected with miR-142-5p/inhibitor/miR-NC 48 h later.

We tested the ability of miR-142-5p regulating the four putative target genes using luciferase reporter assays. As a result, the luminecence intensities of SMAD3 transfected were significantly decreased compared with the control groups (Figure 3B and Supplementary Figure S4), suggesting that miR-142-5p can bind to the 3′ UTRs of *SMAD3*. Furthermore, we also validated the results with the mutant *SMAD3* 3′ UTRs by luciferase assays and found that the mutant binding sites were not bound by miR-142-5p (Figure 3A–3B). These data demonstrated that miR-142-5p binds to the 3′ UTRs of *SMAD3* on the predicted binding sites.

Then, we further investigated whether the binding of miR-142-5p to *SMAD3* will result in their down-regulation of expression or not. We transfected human lung adenocarcinoma (A549) and breast cancer (MDA-MB-231) cells with miR-142-5p mimics and inhibitors to test the protein level of targets by western blotting. Immunoblotting assay results showed that miR-142-5p overexpression caused an apparent decrease on the levels of SMAD3 proteins in A549 and MDA-MB-231 cells, whereas inhibition of miR-142-5p had the opposite effect (Figure 3C). Therefore, these data supported that miR-142-5p directly associated with the mRNA 3′UTR region of SMAD3 transcript, thereby identifying SMAD3 as an authentic target of miR-142-5p.

### miR-142-5p promotes cell proliferation by attenuating TGF-β mediated inhibitory effects

**Figure 4:**
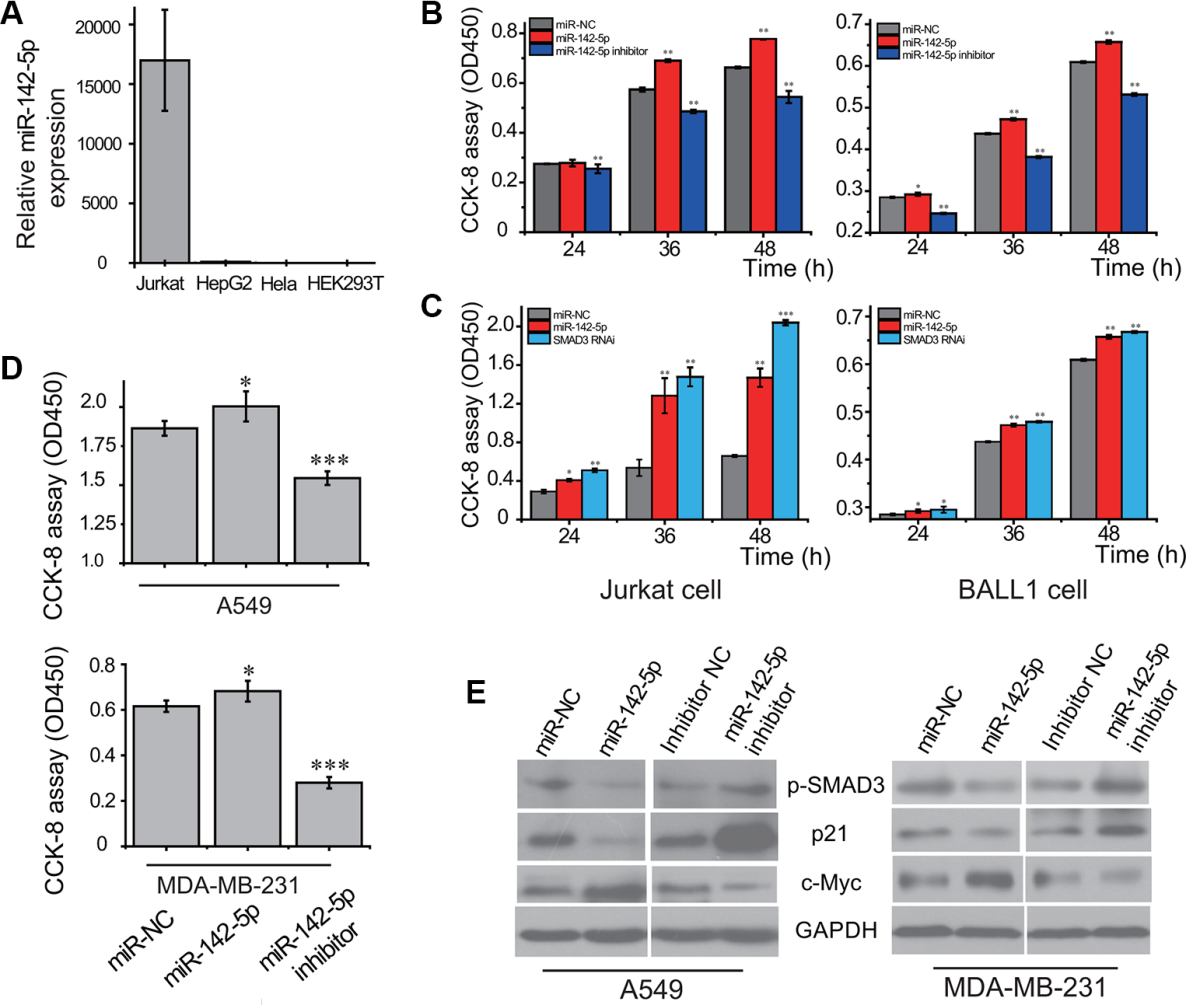
miR-142-5p promotes the proliferation of tumor cells by regulating TGF-β pathway (**A**) The expression of miR-142-5p in 4 cell lines (Jurkat, HepG2, HeLa and HEK293T) detected by real-time RT-PCR. 5S RNA was used as the internal control. (**B**) miR-142-5p promotes the proliferation of Jurkat and BALL-1 cells transfected with miR-142-5p/inhibitor/miR-NC. (**C**) Knockdown of SMAD3 promotes cell proliferation of Jurkat and BALL-1 cells, resembling the effect of overexpressing miR-142-5p. Viable cells were detected using Cell Counting Kit-8 (CCK-8 kit). (**D**) miR-142-5p promotes the proliferation of A549 and MDA-MB-231 cells transfected with miR-142-5p/inhibitor/miR-NC 36 h later. (**E**) Immunoblotting assays were used to analyze the expressions of SMAD3 phosphorylation (p-SMAD3), p21 and c-Myc protein levels in A549 and MDA-MB-231 cell lines. Data represent means and s.e of three experiments performed in triplicate and normalized to control RNA. Data in histograms are represented as mean ± SD. *T* test, *P < 0.05, **P < 0.01, ***P < 0.001 compared with the control.

To explore the potential role of miR-142-5p in cancer, we performed experiments to analyze the expression of miR-142-5p in human cancer cell lines by real-time RT-PCR. As shown in Figure 4A, a strong expression of miR-142-5p was detected in human acute leukemic Jurkat cell line, but not in human hepatocarcinoma HepG2, cervical carcinoma HeLa or embryonic kidney 293T cell lines. Moreover, another analyses showed miR-142-5p was significantly up-regulated in lung adenocarcinoma and breast cancer tissues compared to normal tissues in TCGA (Supplementary Figure S5) and HMED (http://bioinfo.life.hust.edu.cn/smallRNA/index.php) databases [30]. These findings suggested the potential role of miR-142-5p as a tumor regulator in human cancers.

We next examined the effects of miR-142-5p on cell proliferation and apoptosis in several cancer cells. The cell proliferations of acute leukemia cell lines were increased by overexpression of miR-142-5p, but reduced when miR-142-5p expression was silenced (Figure 4B). Consistent with above assays, we found that miR-142-5p had the similar effect on lung adenocarcinoma and breast cancer cells (Figure 4D). These results indicated that miR-142-5p can regulate the proliferation-related signaling to promote proliferation of cancer cells. Moreover, the effect of miR-142-5p on cell proliferation was further assessed by cell cycle analysis of cervical cancer cells. Overexpression of miR-142-5p caused an increased cell populations at S phase and concomitantly a reduction of cell populations at G0/G1 phase (Supplementary Figure S6), suggesting that miR-142-5p promotes cell cycle progression of Hela cells. Furthermore, the apoptosis assays indicated that overexpression of miR-142-5p inhibited breast cancer cell apoptosis but knockdown of miR-142-5p had the opposite effect (Supplementary Figure S7). Collectively, we validated the oncogenic function of miR-142-5p in several cancer cells.

Inhibition of SMAD3 expression by miR-142-5p could, in principle, diminish the dependence of the canonical TGF-β signaling pathway. Thus, we further evaluated the effects of SMAD3 using cells transfected with RNAi manipulation. The knockdown of SMAD3 led to significant promotion of Jurkat and BALL-1 cell proliferation, resembling the effect of overexpression of miR-142-5p (Figure 4C). These results suggest that the attenuation of canonical TGF-β signaling pathway and promotion of cell proliferation by miR-142-5p is mostly mediated by the ability of miR-142-5p to reduce the protein level of SMAD3. Phosphorylation and subsequent translocation of SMAD3 to the nucleus are critical steps in TGF-β signal transduction [16]. Therefore, the effect of miR-142-5p in the attenuation of the TGF-β pathway was examined in cancer cells. Western blotting revealed that the phosphorylation of SMAD3 was reduced by overexpression of miR-142-5p, but increased when miR-142-5p expression was silenced (Figure 4D). To further confirm our results, we tested the downstream signaling pathway of TGF-β. Overexpression of miR-142-5p had significantly downregulated p21, and upregulated c-Myc protein levels, whereas knockdown of miR-142-5p had the opposite effect (Figure 4D). The results demonstrated that miR-142-5p suppressed TGF-β-induced growth inhibition in cancer cells.

## DISCUSSION

In this study, we performed a miRNA regulatory pathway inference and identified cancer-related miRNA regulatory pathway networks by integrating miRNAs/genes expression in cancers. We predicted and validated miR-142-5p as a negative regulator of TGF-β signaling pathway by targeting SMAD3. These findings suggest that miR-142-5p may be critical in cancers and serve as a potential therapeutic target.

MiRNAs have emerged as crucial mediators of human diseases by targeting multitudinous genes and affecting gene regulation networks. MiRNA expression profiles classify human cancers and improve our understanding of the heterogeneity [31, 32]. Thus, the identification of tumor-related network of miRNAs and pathways is critical for understanding the roles of miRNAs in tumorigenesis. MiRNAs play a fine-tuning role in gene expression, the integrating miRNA-expression data will be more biologically meaningful to explore the disease-related network. By integration of miRNA regulatory pathway inference and miRNA/gene expression data, we found and experimentally confirmed that miR-142-5p attenuates TGF-β signaling pathway in tumor cells, suggesting a feasible approach with bioinformatics guidance in studying disease-related regulatory networks.

We observed that miR-142-5p promoted the growth and survival of human cancer cells by suppressing TGF-β signaling. A latest research reported that up-regulated miR-142-5p inhibits TGF-β-induced apoptosis via targeting TGFBR2 and SMAD3 in rotavirus infection model [33]. Our studies indicated that miR-142-5p overexpression significantly downregulated SMAD3 to diminish TGF-β-induced growth arrest, supporting Chanda et al. research from several tumor cell lines. Recent researches have indicated a controversial function for miR-142-5p in cell growth and cell progression. MiRNA profiling of clinical samples and cell lines has shown that miR-142-5p is upregulated in vascular smooth muscle cells [34], and downregulated in renal cell carcinoma and systemic lupus erythematosus [35–37], indicating that miR-142-5p could suppress cell growth or promotes cell proliferation in different conditions. Our investigations indicated that miR-142-5p is highly and differentially expressed in some tumor cells, indicating that miR-142-5p functions an oncogenic role by suppressing the translation of SMAD3 to promote cancer cell growth.

TGF-β signaling is known to play a complicated role in tumorigenesis. There are growing evidences demonstrating that miRNAs are closely associated with the TGF-β signaling pathway in various tumor types [22]. In this study, we identified miR-142-5p as a negative regulator of TGF-β signaling pathway in human cancer cells. The miR-142 gene was found at the breakpoint junction of a t(8;17) translocation, which causes an aggressive B cell leukemia due to strong upregulation of a translocated MYC gene [38]. Whereas the TGF-β pathway suppresses proliferation by down-regulating MYC and the cyclin-dependent kinase inhibitors, MYC modulate biosynthesis of miRNA-101 and miR-17 cluster in the nucleus [39, 40]. We analyzed the upstream sequence of the miR-142 gene and also found a MYC-binding site in its promoter, suggesting that MYC regulates the biosynthesis of miR-142 precursors. Collectively, our and other studies indicated that miR-142-5p/3p attenuate TGF-β signal transduction by targeting TGFBRI/II and SMAD3 [33, 41]. A combinatorial miRNA-TF feedback loop could be constructed to modulate the TGF-β signaling. In summary, miR-142 functions as a versatile regulator to modulate TGF-β signaling network by targeting key signaling components.

In conclusion, this work studied the miRNA regulated pathways, identified some cancer-related miRNA networks and further experimentally validated a key miRNA pathway regulation. The results expanded our understanding of the mechanisms underlying the post-transcriptional regulation of Smad complex to mediate the canonical TGF-β signaling. This study provided a feasible systems biology approach to study the function of miRNAs in complex diseases.

## MATERIALS AND METHODS

### MiRNA target genes, pathway genes data sources and process

To collect miRNAs and their potential target genes, we firstly compiled seven online databases (TargetScan, miRanda, TarBase, miR2Disease, miRTarBase, miRecords, miRWalk) with predicted targets or experimentally validated targets of miRNAs (Supplementary Figure S1). We intersected predicted miRNA targets from TargetScan and miRanda, and unioned verified miRNA targets from TarBase, miRTarBase, miR2Disease, miRecords, miRwalk to get the final miRNA-target result as our previous studies [42, 43]. Gene sets of signaling pathways were obtained from the Cancer Genome Anatomy Project database (http://cgap.nci.nih.gov/Pathways/BioCarta_Pathways). And we applied Fisher's exact test to identify the miRNA-pathway enrichment results and obtain the *P*-value (Supplementary Table S1).

### TCGA expression data process and miRNA pathway enrichment analysis

To get the TCGA mRNA and miRNA expression data, we obtained the miRNA and mRNA expression profiles for 20 TCGA tumor types with both case and control data from Firebrowse (http://firebrowse.org/). And we obtained the average miRNA expression RPM (TCGA normalized miRNA expression) and mRNA expression RSEM (TCGA normalized mRNA expression) in case/normal samples of each cancer to perform differentially expression analysis. The differentially expressed miRNAs and mRNAs (case or normal RPM/RSEM > 50, case/normal fold change > 2 or < 0.5) were filtered for further analysis. Then, the collected miRNA target pairs with a negative correlation between miRNA and mRNA were considered as true miRNA-target pairs. We applied one-tail Fisher's exact test to explore the enrichment analysis of miRNA target genes in each BioCarta pathway and tumor type.

### Cell lines and cell culture

Human ALL cell lines Jurkat and BALL-1 were grown in RPMI-1640, containing 10% fetal bovine serum and 100 mg/ml penicillin/streptomycin. Human solid tumor cell lines (HepG2, Hela, A549, and MDA-MB-231) and HEK-293T cells were cultured in Dulbecco's modified Eagle's medium, containing 10% fetal bovine serum and 100 mg/ml penicillin/streptomycin. All the cells were grown in an atmosphere of 5% CO_2_ at 37°C.

### RNA oligoribonucleotides and transient transfections

MiR-142-5p (5′-CAUAAAGUAGAAAGCACU ACU-3′) duplex, miR-142-5p specific inhibitor (5′-AGUAGUGCUUUCUACUUUAUG-3′) molecules and appropriate negative control molecules were purchased from RiboBio Corporation (Guangzhou, China). Small interfering RNA (siRNA) duplexes and the corresponding scrambled control oligonucleotides were synthesized from Genephama Biotech (Shanghai, China). siRNA oligonucleotides against human SMAD3 (GenBank access no: NM_001145102) are GGAGAAAUGGUGCGAGAAGdTdT (Forward) and CUUCUCGCACCAUUUCUCCdTdT (Reverse). Transient transfection of RNA oligoribonucleotides was performed using X-tremeGENE siRNA Transfection Reagent (Roche, Germany) according to the manufacturer's protocol. The final concentration was 125 nM for miRNA mimics or 60 nM for siRNA, and 250 nM for miRNA inhibitors.

### Analysis of miRNA expression by real-time RT-PCR

Cells were lysed with TRIzol reagent (Invitrogen, Carlsbad, CA, USA), and total RNA was extracted according to the manufacturer's instructions. For mature miRNA expression analysis, miR-142-5p (5′-CAUAAAGUAGAAAGCACUACU-3′) was detected using an All-in-One™ miRNA qRT-PCR Detection kit (GeneCopoeia™, Guangzhou, China) according to the manufacturer's instructions. The 5S rRNA was used as an internal control for miR-142-5p. All reactions were performed in triplicate and at least three times independently.

### Cell proliferation assay

For quantitative analysis of the cell proliferation rate, 10 μL of the CCK-8 Kit (Dojindo, Kumamoto, Japan) solution was added to each well. After incubation at 37°C for 4 h in a humidified CO_2_ incubator, absorbance at 450 nm was monitored with a microplate reader (Multiskan MK3, Thermo Lab systems, Shanghai, China). The obtained values were normalized to those from control cells transfected with scramble oligonucleotides. All experiments were performed in triplicates.

### Cell cycle and apoptosis analysis

The Hela cells were fixed with 75% ethanol at 4°C overnight and washed with phosphate-buffered saline and treated with RNaseI, followed by staining with propidium iodide for 30 min. Cell cycle analysis was performed by flow cytometry (FACSCalibur, Becton Dicson). Apoptotic cells were stained using Annexin V-FITC Apoptosis Detection Kit I (BD Biosciences) and analysed by flow cytometry following the manufacturer's instruction.

### Luciferase assays

The fragments of the 3′UTR of the predicted targets containing the target sequence of miR-142-5p were amplified by RT-PCR. Primers used for amplification of specific cDNA probes are given in the Supplementary Table S3. The fragments were inserted into the psi-CHECK2 vector (XhoI and NotI restriction enzyme sites; Promega). HEK-293T cells were transfected with psiCHECK/mRNA-3′UTR construct using Lipofectamine 2000 (Invitrogen, USA) and the appropriate miRNA or negative control. Luciferase and renilla signals were measured 48 h after transfection according to the manufacturer's instructions and our previous description [44].

### Western blotting assays

Western blotting analysis was performed according to a standard method, as described previously [44], using anti–SMAD3, anti–Phospho-Smad3 (Ser423/425), anti–p21 Waf1/Cip1 and anti–c-MYC (Cell Signaling Tech, USA). Following the initial western blot assay, the membranes were stripped and re-probed with anti-GAPDH (Tianjin Sungene Biotech Co., China) as a protein loading control.

## SUPPLEMENTARY MATERIALS FIGURES AND TABLES


