# Supplementary material for: MicroRNA regulatory pathway analysis identifies miR-142-5p as a negative regulator of TGF-β pathway via targeting SMAD3
Source: Oncotarget. 2016 Sep 24;7(44):71504–13. doi: 10.18632/oncotarget.12229 (PMC5342096; doi:10.18632/oncotarget.12229)
Supplement: Supplementary file 1 [file oncotarget-07-71504-s001.pdf]

MicroRNA regulatory pathway analysis identifies miR-142-5p as a negative regulator of TGF-β pathway via targeting SMAD3

Supplementary Materials

Supplementary Table S1: miRNA pathway regulation numbers in each tumor types

Tumor type	20 tumor with both case and normal samples miR-pathway numbers
BLCA	2781
BRCA	1112
CESC	2240
CHOL	1005
ESCA	742
HNSC	759
KICH	540
KIRC	545
KIRP	530
LIHC	952
LUAD	999
LUSC	926
PAAD	708
PCPG	1486
PRAD	677
SKCM	1719
STAD	818
THCA	613
THYM	971
UCEC	2219

Supplementary Table S2: One tail fisher's exact test analysis

	In Pathway N	Not in Pathway N	Total
In M target set	a+	a−	a
Not in M target set	b+	b−	b
Total	c+	c−	c

a: number of miRNA M targeted nodes. b: number of miRNA M non-target nodes. c+: number of nodes in pathway N. c−: number of nodes not in pathway N. a+: number of targeted nodes in pathway N. a−: number of target nodes not in pathway N. b+: number of non-targeted nodes in pathway N. b−: number of non-targeted nodes not in pathway N. c: all nodes.

Supplementary Table S3: The primers for amplification of specific cDNAs

Predicted targets	Chain	PCR primers	Product size (bp)
SMAD3	Forward	cacaactcgagCAGCTCTCTGACGCTTGTGACAGTG	1463
	Reverse	aagcggccgcAGGCACATACTTCAGACTCACAGCAT	
TGFBR1	Forward	cacaactcgagGGAGGTCAATTGTTCTACCTCACTGAGA	2536
	Reverse	aagcggccgcTAGGATGGGCAGTATAAACAACTGTG	
TGFBR2	Forward	cacaactcgagCCAAAGAACAGAGGCAGCAGGAA	1563
	Reverse	aagcggccgcGTAAAGCACTCCTCTCTCCAATGCAGA	
TGFB2	Forward	cacaactcgagCCTTCCCATTCTTACTCTT	1050
	Reverse	aagcggccgcGAACCCTAAATCCTTGCT	

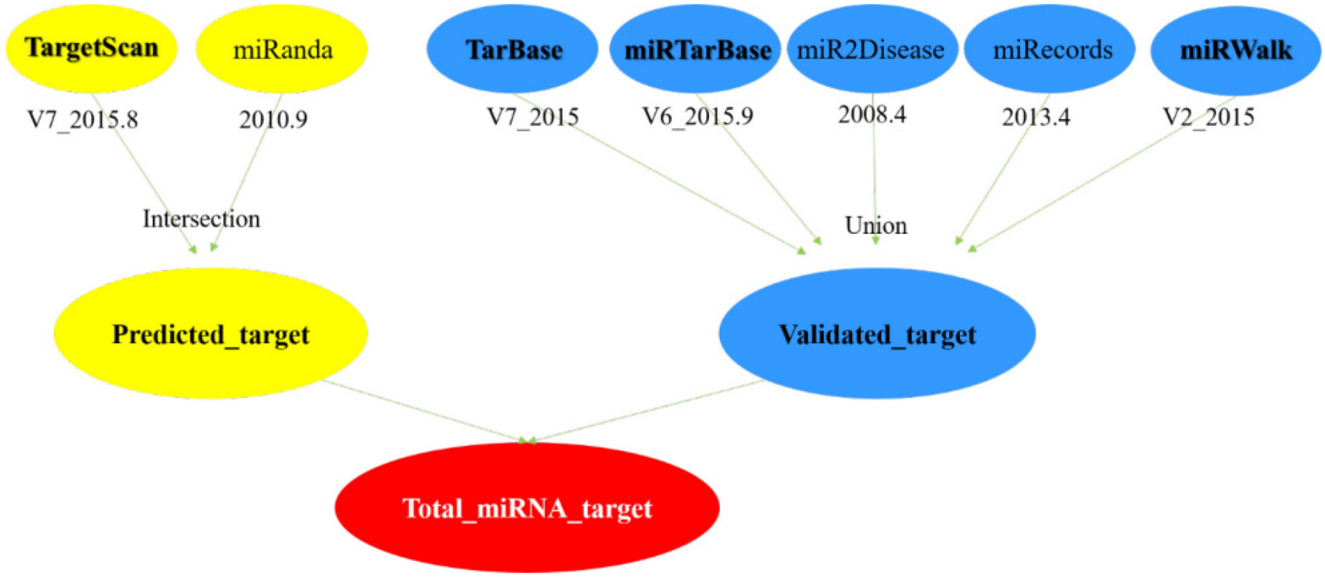

Supplementary Figure S1: MiRNA data source and process. we compiled seven online databases (TargetScan, miRanda, TarBase, miR2Disease, miRTarBase, miRecords, miRWalk) with predicted targets or experimentally validated targets of miRNAs. We intersected predicted miRNA targets from TargetScan and miRanda, and unioned verified miRNA targets from TarBase, miRTarBase, miR2Disease, miRecords, miRwalk to get the final miRNA-target result.

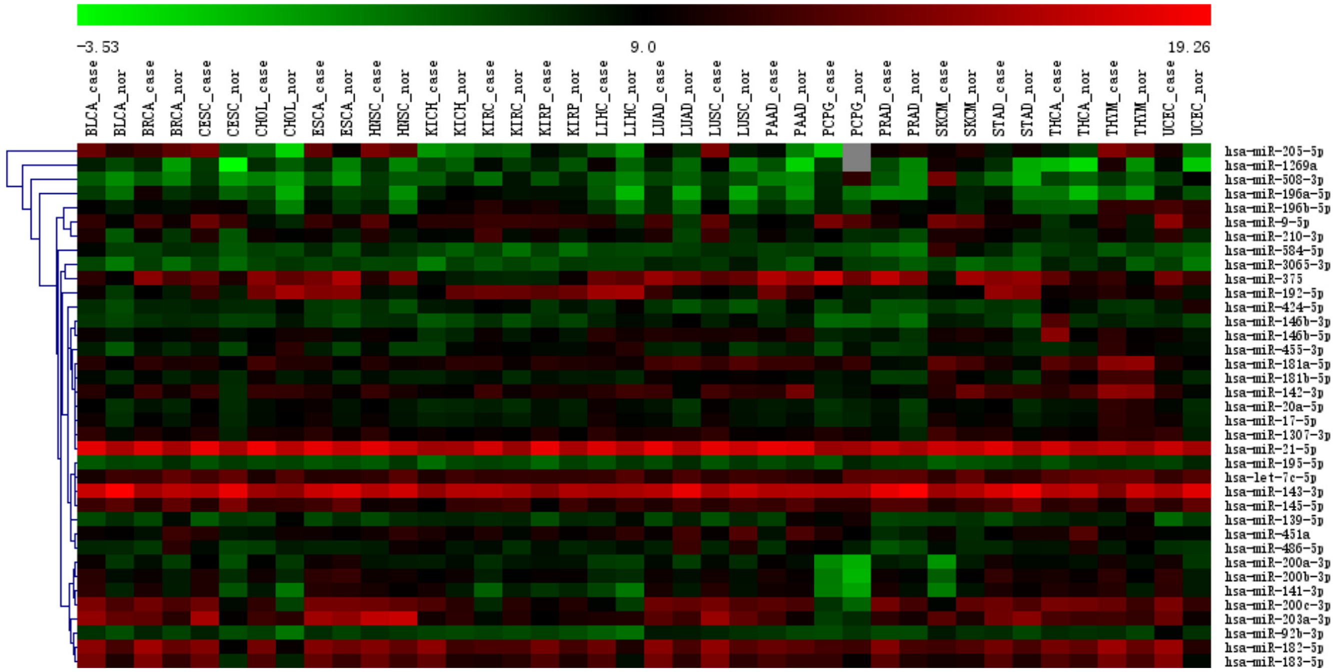

Supplementary Figure S2: Heatmap of 37 miRNAS expressed differently more than 10 tumer types. Expression of these miRNAs transformed to log2 RSEM.

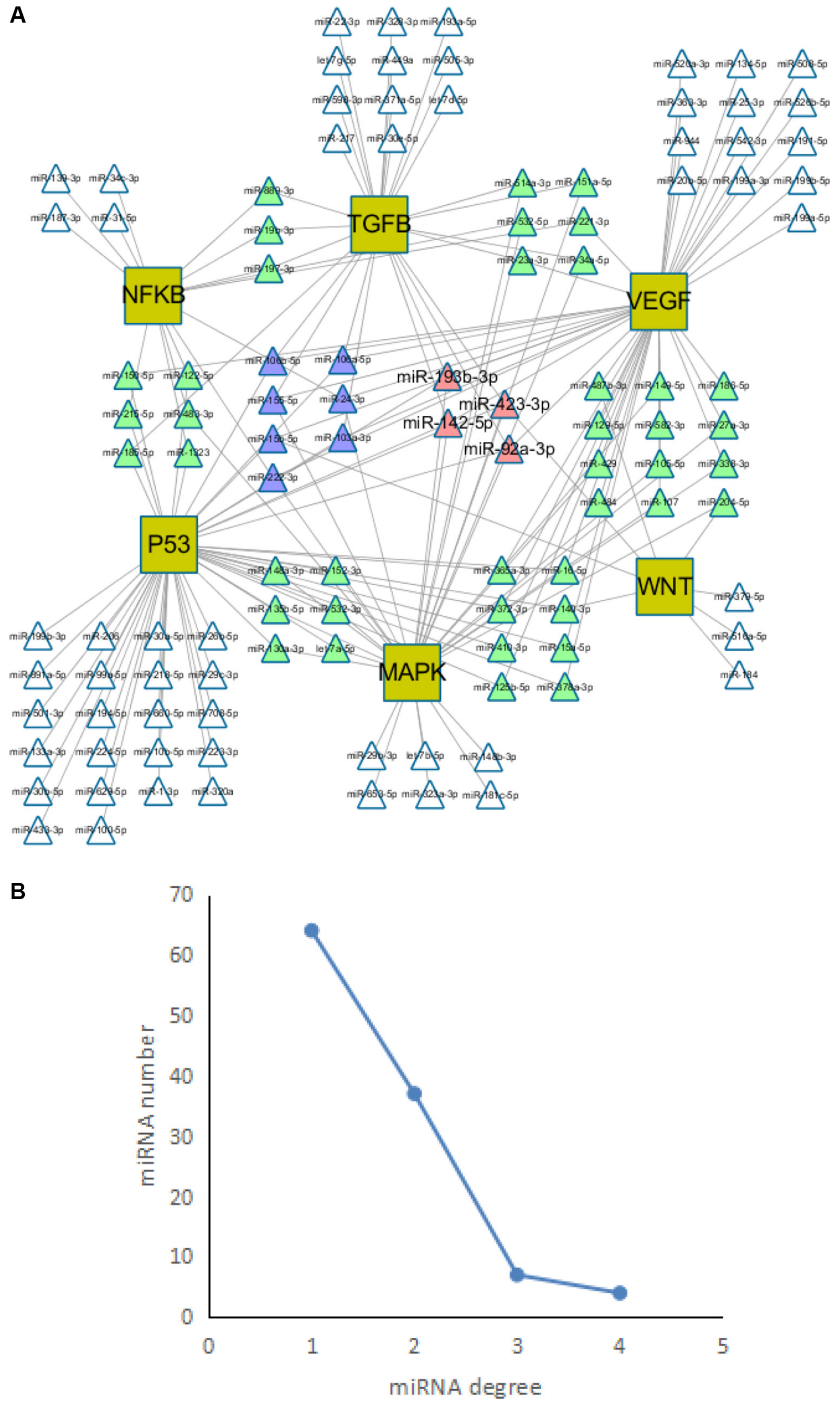

Supplementary Figure S3: (**A**) The network of six cancer related pathways regulated by miRNAs that differentially expressed in less than 10 tumor types. The white, green, blue and red triangles were miRNAs that regulated one, two, three and four pathways, respectively. (**B**) Statistics of miRNA degree in miRNA pathway networks.

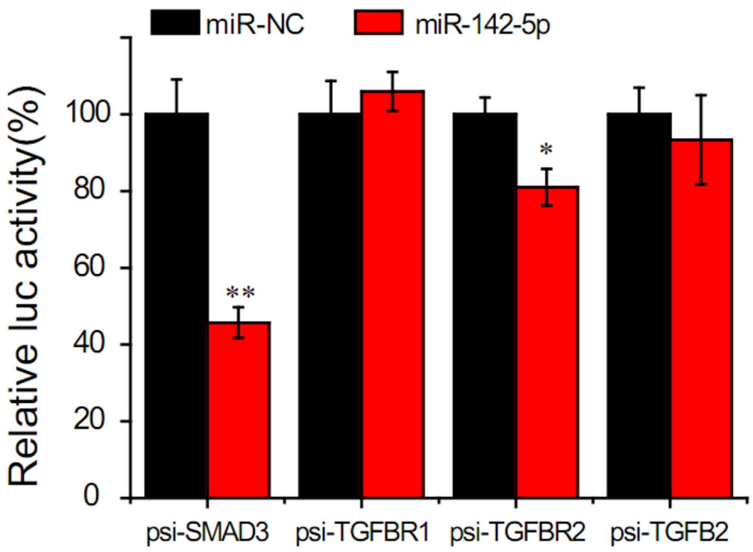

Supplementary Figure S4: The luminecence intensities of predicted targets in the TGF-β pathway. Firefly and Renilla luciferase activities were measured as described in materials and methods.

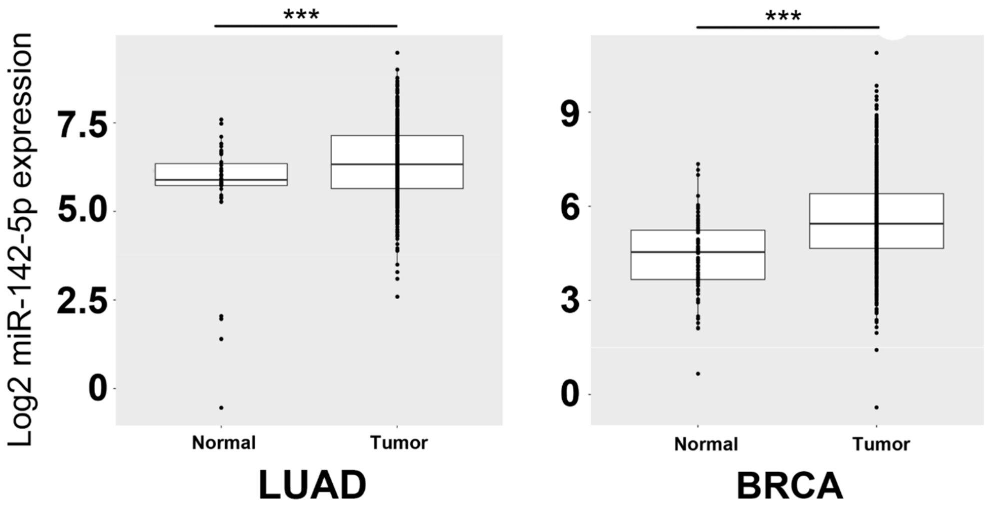

Supplementary Figure S5: Expression of miR-142-5p was up-regulated in LUAD (A) and BRCA (B) tissues compared to normal tissues in TCGA. ***$P < 0.001$. LUAD: Lung adenocarcinoma, BRCA: Breast invasive carcinoma.

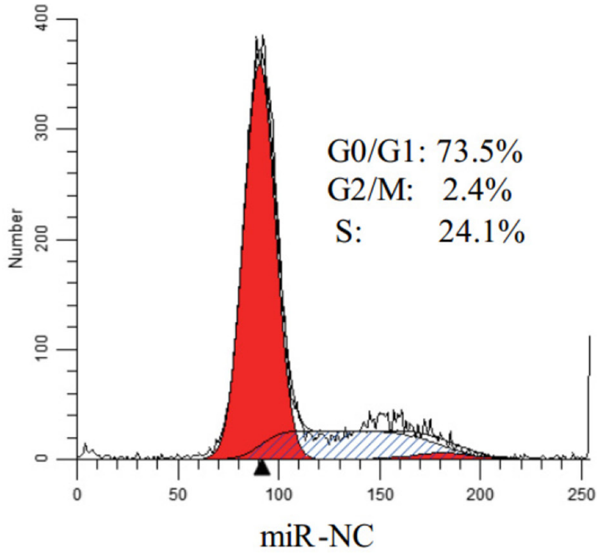

Supplementary Figure S6: miR-142-5p overexpression promotes cell cycle progression in Hela cells. miR-142-5p mimics and control RNAs were transfected into Hela cells. 24 h later, cell cycle distribution was studied by flow cytometry as described in materials and methods.

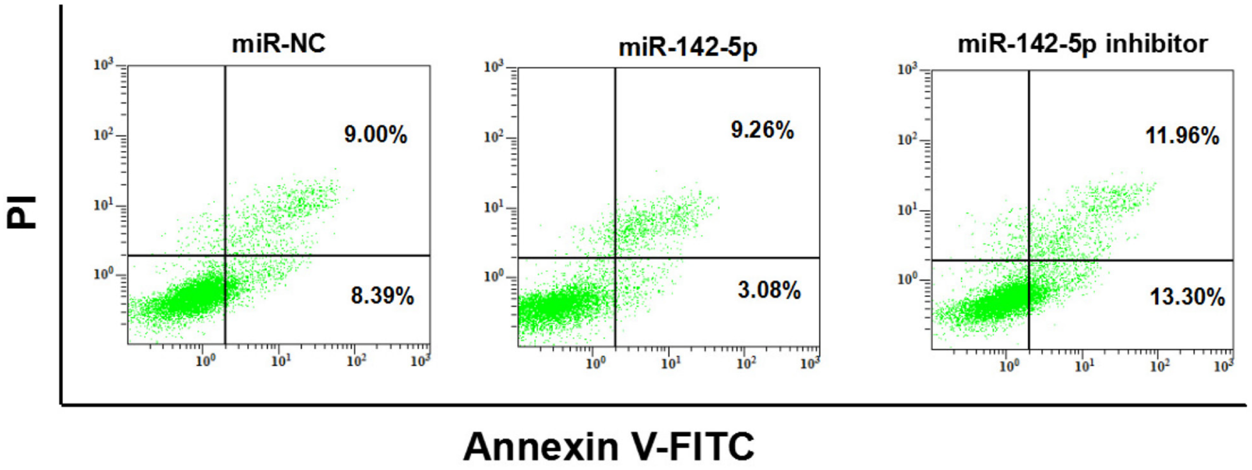

Supplementary Figure S7: Ectopic expression of miR-142-5p attenuates cell apoptosis. The MDA-MB-231 cells were transfected with miR-142-5p/inhibitor/miR-NC 48h later, apoptosis cells were stained with Annexin V-FITC/PI and analyzed. The percentage at the upper right (UR) area and lower right (LR) area stands for the total apoptosis (UR + LR).
